# Hypothesis-driven genome-wide association studies provide novel insights into genetics of reading disabilities

**DOI:** 10.1038/s41398-022-02250-z

**Published:** 2022-11-29

**Authors:** Kaitlyn M. Price, Karen G. Wigg, Else Eising, Yu Feng, Kirsten Blokland, Margaret Wilkinson, Elizabeth N. Kerr, Sharon L. Guger, Quantitative Trait Working Group of the GenLang Consortium, Filippo Abbondanza, Andrea G. Allegrini, Till F. M. Andlauer, Timothy C. Bates, Manon Bernard, Milene Bonte, Dorret I. Boomsma, Thomas Bourgeron, Daniel Brandeis, Manuel Carreiras, Fabiola Ceroni, Valéria Csépe, Philip S. Dale, John C. DeFries, Peter F. de Jong, Jean Francois Démonet, Eveline L. de Zeeuw, Marie-Christine J. Franken, Clyde Francks, Margot Gerritse, Alessandro Gialluisi, Scott D. Gordon, Jeffrey R. Gruen, Marianna E. Hayiou-Thomas, Juan Hernández-Cabrera, Jouke-Jan Hottenga, Charles Hulme, Philip R. Jansen, Juha Kere, Tanner Koomar, Karin Landerl, Gabriel T. Leonard, Zhijie Liao, Michelle Luciano, Heikki Lyytinen, Nicholas G. Martin, Angela Martinelli, Urs Maurer, Jacob J. Michaelson, Nazanin Mirza-Schreiber, Kristina Moll, Anthony P. Monaco, Angela T. Morgan, Bertram Müller-Myhsok, Dianne F. Newbury, Markus M. Nöthen, Richard K. Olson, Silvia Paracchini, Tomas Paus, Zdenka Pausova, Craig E. Pennell, Bruce F. Pennington, Robert J. Plomin, Franck Ramus, Sheena Reilly, Louis Richer, Kaili Rimfeld, Gerd Schulte-Körne, Chin Yang Shapland, Nuala H. Simpson, Shelley D. Smith, Margaret J. Snowling, Beate St Pourcain, John F. Stein, Joel B. Talcott, Henning Tiemeier, J. Bruce Tomblin, Dongnhu T. Truong, Elsje van Bergen, Marc P. van der Schroeff, Marjolein Van Donkelaar, Ellen Verhoef, Carol A. Wang, Kate E. Watkins, Andrew J. O. Whitehouse, Erik G. Willcutt, Margaret J. Wright, Gu Zhu, Simon E. Fisher, Maureen W. Lovett, Lisa J. Strug, Cathy L. Barr

**Affiliations:** 1 Division of Experimental and Translational Neuroscience, Krembil Research Institute, University Health Network, Toronto, Ontario Canada; 2 Program in Neuroscience and Mental Health, Hospital for Sick Children, Toronto, Ontario Canada; 3 Department of Physiology, University of Toronto, Toronto, Ontario Canada; 4 Language and Genetics Department, Max Planck Institute for Psycholinguistics, Nijmegen, The Netherlands; 5 Department of Psychology, Hospital for Sick Children, Toronto, Ontario Canada; 6 Department of Pediatrics, University of Toronto, Toronto, Ontario Canada; 7 Donders Institute for Brain, Cognition and Behaviour, Radboud University, Nijmegen, The Netherlands; 8 Genetics and Genome Biology, Hospital for Sick Children, Toronto, Ontario Canada; 9 Departments of Statistical Sciences and Computer Science, Faculty of Arts and Science and Division of Biostatistics, Dalla Lana School of Public Health, University of Toronto, Toronto, Ontario Canada; 10 School of Medicine, University of St Andrews, KY16 9TF St. Andrews, Scotland; 11 Social, Genetic and Developmental Psychiatry Centre, Institute of Psychiatry, Psychology and Neuroscience, King’s College London, London, UK; 12 Translational Research in Psychiatry, Max Planck Institute of Psychiatry, Munich, Germany; 13 Department of Neurology, Klinikum rechts der Isar, School of Medicine, Technical University of Munich, Munich, Germany; 14 Department of Psychology, University of Edinburgh, Edinburgh, EH8 9JZ UK; 15 Department of Physiology and Nutritional Sciences, University of Toronto, Toronto, ON M5S 1A1 Canada; 16 Department of Cognitive Neuroscience and Maastricht Brain Imaging Center, Faculty of Psychology and Neuroscience, Maastricht University, Maastricht, The Netherlands; 17 Netherlands Twin Register, Amsterdam, The Netherlands; 18 Department of Biological Psychology, Vrije Universiteit Amsterdam, 1081 BT Amsterdam, The Netherlands; 19 Amsterdam Reproduction and Development (AR&D) Research Institute, Amsterdam, UMC The Netherlands; 20 Human Genetics and Cognitive Function Unit, Institut Pasteur, Paris, France; 21 Centre National Recherche Scientifique (CNRS) UMR 3571, Université de Paris, Paris, France; 22 Department of Child and Adolescent Psychiatry and Psychotherapy, Psychiatric University Hospital Zurich, University of Zurich, 8032 Zurich, Switzerland; 23 Zurich Center for Integrative Human Physiology, University of Zurich and ETH Zurich, 8057 Zurich, Switzerland; 24 Neuroscience Center Zurich, University of Zurich and ETH Zurich, 8057 Zurich, Switzerland; 25 Department of Child and Adolescent Psychiatry and Psychotherapy, Central Institute of Mental Health, Medical Faculty Mannheim, Heidelberg University, 68159 Mannheim, Germany; 26 Basque Center on Cognition, Brain and Language (BCBL), Donostia-San Sebastian, Gipuzkoa Spain; 27 Ikerbasque, Basque Foundation for Science, Bilbao, Vizcaya Spain; 28 Lengua Vasca y Comunicación, University of the Basque Country (UPV/EHU), Bilbao, Vizcaya Spain; 29 Department of Pharmacy and Biotechnology, University of Bologna, 40126 Bologna, Italy; 30 Faculty of Health and Life Sciences, Oxford Brookes University, Oxford, OX3 0BP UK; 31 Brain Imaging Centre, Research Centre for Natural Sciences, Budapest, 1117 Hungary; 32 Multilingualism Doctoral School, Faculty of Modern Philology and Social Sciences, University of Pannonia, Veszprém, 8200 Hungary; 33 Department of Speech & Hearing Sciences, University of New Mexico, Albuquerque, NM 87131 USA; 34 Institute for Behavioral Genetics, University of Colorado, Boulder, CO 80309-0447 USA; 35 Department of Psychology and Neuroscience, University of Colorado, Boulder, CO 80309-0447 USA; 36 Department of Child Development and Education, University of Amsterdam, 1012 WX Amsterdam, The Netherlands; 37 Leenaards Memory Centre, Department of Clinical Neurosciences, Lausanne University Hospital (CHUV), University of Lausanne, CH-1011 Lausanne, Switzerland; 38 Department of Otolaryngology, Head and Neck Surgery, Erasmus University Medical Center, 3015 GD Rotterdam, The Netherlands; 39 Department of Human Genetics, Radboud University Medical Center, 6525 GA Nijmegen, The Netherlands; 40 Department of Epidemiology and Prevention, IRCCS Istituto Neurologico Mediterraneo Neuromed, Pozzilli, Italy; 41 EPIMED Research Center, Department of Medicine and Surgery, University of Insubria, Varese, Italy; 42 Genetic Epidemiology Laboratory, QIMR Berghofer Medical Research Institute, Brisbane, QLD 4006 Australia; 43 Departments of Pediatrics and Genetics, Yale Medical School, New Haven, CT 06510 USA; 44 Department of Psychology, University of York, York, YO10 5DD UK; 45 Departamento de Psicología, Clínica Psicobiología y Metodología, 38200 La Laguna, Santa Cruz de Tenerife, Spain; 46 Department of Education, University of Oxford, Oxford, Oxfordshire OX2 6PY UK; 47 Department of Child and Adolescent Psychiatry/Psychology, Erasmus University Medical Center, 3000 CB Rotterdam, The Netherlands; 48 Department of Complex Trait Genetics, Center for Neurogenomics and Cognitive Research, Amsterdam Neuroscience, VU University, Amsterdam, The Netherlands; 49 Department of Human Genetics, VU Medical Center, Amsterdam University Medical Center, 1081 BT Amsterdam, The Netherlands; 50 Department of Biosciences and Nutrition, Karolinska Institutet, 171 77 Stockholm, Sweden; 51 Stem Cells and Metabolism Research Program, University of Helsinki and Folkhälsan Research Center, 00014 Helsinki, Finland; 52 Department of Psychiatry, University of Iowa, Iowa City, IA 52242 USA; 53 Institute of Psychology, Karl-Franzens-Universität Graz, 8010 Graz, Austria; 54 BioTechMed-Graz, Graz, Austria; 55 Cognitive Neuroscience Neurology and Neurosurgery, McGill University, Montreal, QC H3A 1G1 Canada; 56 Department of Psychology, University of Toronto, Toronto, ON Canada; 57 Department of Psychology, University of Jyväskylä, 40014 Jyväskylä, Finland; 58 Department of Psychology, The Chinese University of Hong Kong, Hong Kong, China; 59 Institute of Neurogenomics, Helmholtz Zentrum München, Munich, Germany; 60 Department of Child and Adolescent Psychiatry, Psychosomatics, and Psychotherapy, Ludwig-Maximilians-University Hospital Munich, Munich, 80336 Germany; 61 Office of the President, Tufts University, Medford, MA 02155 USA; 62 Speech and Language, Murdoch Children’s Research Institute, Melbourne, VIC 3052 Australia; 63 Department of Audiology and Speech Pathology, University of Melbourne, Melbourne, VIC 3052 Australia; 64 Speech Pathology Department, Royal Children’s Hospital, Melbourne, VIC 3052 Australia; 65 Department of Health Science, University of Liverpool, Liverpool, L69 7ZX UK; 66 Institute of Human Genetics, University Hospital of Bonn, 53127 Bonn, Germany; 67 Department of Psychiatry and Neuroscience and Centre Hospitalier Universitaire Sainte Justine, University of Montreal, Montreal, QC H3T 1J4 Canada; 68 Department of Psychiatry, University of Toronto, Toronto, ON Canada; 69 Department of Psychology, University of Toronto, Toronto, ON Canada; 70 Translational Medicine, Hospital for Sick Children, Toronto, ON Canada; 71 School of Medicine and Public Health, College of Health, Medicine and Wellbeing, University of Newcastle, Newcastle, NSW 2308 Australia; 72 Mothers and Babies Research Program, Hunter Medical Research Institute, Newcastle, NSW 2305 Australia; 73 Maternity and Gynaecology, John Hunter Hospital, Newcastle, New South Wales Australia; 74 Department of Psychology, University of Denver, Denver, CO USA; 75 Laboratoire de Sciences Cognitives et Psycholinguistique, Ecole Normale Supérieure, Paris Sciences & Lettres University, École des Hautes Études en Sciences Sociales (EHESS), Centre National de la Recherche Scientifique (CNRS), Paris, 75005 France; 76 The Health Group, Griffith University, Gold Coast, Queensland Australia; 77 Department of Health Sciences, Université du Québec à Chicoutimi, Chicoutimi, QC Canada; 78 Department of Psychology, Royal Holloway, University of London, Egham, TW20 0EY UK; 79 MRC Integrative Epidemiology Unit, University of Bristol, Bristol, BS8 2BN UK; 80 Population Health Sciences, University of Bristol, Bristol, BS8 2PS UK; 81 Department of Experimental Psychology, University of Oxford, Oxford, OX2 6GG UK; 82 Department of Neurological Sciences, College of Medicine, University of Nebraska Medical Center, Omaha, NE 68198 USA; 83 St John’s College, University of Oxford, Oxford, OX1 3JP UK; 84 Department of Physiology, Anatomy and Genetics, Oxford University, Oxford, OX1 3PT UK; 85 Institute of Health and Neurodevelopment, Aston University, Birmingham, B4 7ET UK; 86 T.H. Chan School of Public Health, Harvard, Boston, MA 02115 USA; 87 Communication Sciences and Disorders, University of Iowa, Iowa City, IA USA; 88 Research Institute LEARN!, Vrije Universiteit Amsterdam, Amsterdam, The Netherlands; 89 Generation R Study Group, Erasmus MC, Rotterdam, The Netherlands; 90 Telethon Kids Institute, The University of Western Australia, Perth, WA 6009 Australia; 91 Queensland Brain Institute, University of Queensland, Brisbane, Queensland Australia

**Keywords:** Genomics, Learning and memory

## Abstract

Reading Disability (RD) is often characterized by difficulties in the phonology of the language. While the molecular mechanisms underlying it are largely undetermined, loci are being revealed by genome-wide association studies (GWAS). In a previous GWAS for word reading (Price, 2020), we observed that top single-nucleotide polymorphisms (SNPs) were located near to or in genes involved in neuronal migration/axon guidance (NM/AG) or loci implicated in autism spectrum disorder (ASD). A prominent theory of RD etiology posits that it involves disturbed neuronal migration, while potential links between RD-ASD have not been extensively investigated. To improve power to identify associated loci, we up-weighted variants involved in NM/AG or ASD, separately, and performed a new Hypothesis-Driven (HD)–GWAS. The approach was applied to a Toronto RD sample and a meta-analysis of the GenLang Consortium. For the Toronto sample (*n* = 624), no SNPs reached significance; however, by gene-set analysis, the joint contribution of ASD-related genes passed the threshold (*p*~1.45 × 10^–2^, threshold = 2.5 × 10^–2^). For the GenLang Cohort (*n* = 26,558), SNPs in *DOCK7* and *CDH4* showed significant association for the NM/AG hypothesis (sFDR q = 1.02 × 10^–2^). To make the GenLang dataset more similar to Toronto, we repeated the analysis restricting to samples selected for reading/language deficits (*n* = 4152). In this GenLang selected subset, we found significant association for a locus intergenic between *BTG3*-*C21orf91* for both hypotheses (sFDR q < 9.00 × 10^–4^). This study contributes candidate loci to the genetics of word reading. Data also suggest that, although different variants may be involved, alleles implicated in ASD risk may be found in the same genes as those implicated in word reading. This finding is limited to the Toronto sample suggesting that ascertainment influences genetic associations.

## Introduction

Reading Disability (RD), also known as developmental dyslexia, is a neurodevelopmental disorder affecting children globally. In North America alone, it affects 5–7% of individuals [1–6]. RD is characterized by difficulties with word reading and spelling, despite typical intelligence and motivation to learn [7]. Affected children often have comorbid neurodevelopmental disorders, including language or speech impairments, or attention-deficit/hyperactivity disorder (ADHD) [8]. These factors increase social difficulties, decrease self-esteem, and hinder academic/occupational success [9–12]. RD, therefore, represents a major public health concern.

The genetics and underlying mechanisms of RD are not fully known. Twin and family studies initially demonstrated genetic contributions to RD by examining heritability within families [3, 13]. Researchers went on to identify specific chromosomal regions and genes implicated in RD via linkage analysis followed by fine-mapping association studies. These linked regions and genes were supported, to varying degrees, by independent studies [14] and meta-analyses [15–20]; however, sample sizes were small by current standards yielding low power and elevated risk of false-positive findings. Moreover, some results could not be replicated [16, 21, 22]. Therefore, researchers called into question the robustness of the genes as candidates for involvement in RD.

Despite these caveats, a number of candidate genes identified from linkage/fine-mapping studies (*KIAA0319, KIAA0319L, DCDC2, DNAAF4* (previously called *DYX1C1* and *EKN1*)*,* and *ROBO1*) were linked to neuronal migration, suggesting a potential molecular mechanism (but for a critical review see [23]). These associations were pertinent as previous postmortem brain studies (*n* < 10) found heterotopias and cortical dysplasias, signatures of altered migration, in RD-affected individuals [24, 25]. It was theorized that disrupted neuronal migration (DNM) may be involved in RD etiology [26, 27].

The first evidence to implicate allelic variation of these RD candidate genes in DNM came from studies finding that protein motifs/domains encoded by the genes were predicted to function in migration [28–32]. Further evidence emerged from *in utero* knockdown experiments of the genes (*KIAA0319, KIAA0319L, DCDC2*, and *DNAAF4/DYX1C1*) in the developing brains of mice or rats. When gene expression was reduced, neural cells did not migrate as expected from the lower ventricular zone to the higher cortical plate [33–38]. Instead, most cells remained in the ventricular or intermediate zones, indicating disrupted migration, albeit with different patterns of disruption for different genes [33–38]. Experiments in which the candidate genes were overexpressed also resulted in neural phenotypes, including aberrant neurite outgrowth (*Dcdc2*) [39], delayed radial migration (*Kiaa0319*) [40], and altered axon growth and regeneration (*Kiaa0319*) [41].

There was, however, skepticism regarding the proposed roles of these genes in migration, when independent knockout experiments did not replicate the results of the knockdown studies [23, 40, 42–44]. Discrepancies between knockout and knockdown findings may be due to developmental compensation by altered gene expression [45, 46]. For example, data from *Dcdc2* knockout mice supports partial redundancy with *Dcx*, a member of the same gene family, which functionally compensates for the loss of *Dcdc2* [43]. Overall, it remains unclear at this time whether the candidate genes in question are indeed implicated in RD and if this involves effects on neuronal migration [23].

To further identify genes implicated in RD, and as the available techniques advanced, the field moved away from linkage analysis in families to genome-wide association studies (GWAS) -- a more powerful approach for complex traits in which effect sizes of individual risk variants are small. In the last few years, GWAS for RD, reading performance, and/or reading-related tasks have begun to yield results with genome-wide statistical significance (SNP-based analysis (*p*~10^–8^) [47–50]; gene-based analysis (*p*~10^–6^) [51–53]). Across recent significant and previous non-significant GWAS findings, a number of the top genes are thought to be involved in neuronal migration/axon pathfinding, potentially supporting the DNM hypothesis. For example, in a GWAS by Price et al., 2020, using two samples, the Toronto (*n* = 624) and Philadelphia Neurodevelopmental Cohort (*n* = 4430), the most significant SNP (*p* < 5 × 10^–7^) in the Toronto sample was located in an intron of *ARHGAP23*, a gene involved in actin cytoskeleton polymerization/reorganization, neuronal development, and other growth cone/axon related processes [54]. Across both samples in that study, when top-ranked SNPs, at a less stringent p-value threshold (p~10^–5^), were mapped into or near close proximity genes, additional genes were found to have been previously related to neuronal migration/axon guidance (NM-AG) (as well as growth cone formation which is encompassed within this term). For example, *ASTN2, CNTN, TUBB3, NRCAM, DSCAM, UNC5D*, and *GAP43* [53]. Larger GWAS studies also provided weak support for the DNM hypothesis. A meta-analysis of 22 samples (n~34,000) by the GenLang Consortium, identified genome-wide significant variants in *DOCK7* associated with word reading [47]. *DOCK7* is critical for cortical neurogenesis, axon formation, and neural polarization [55]. In the largest GWAS study to date, analyzing the 23andMe cohort, the authors identified 42 significant loci associated with self-reported dyslexia (n_cases_~51,000) of which genes had been previously related to NM/AG [48] (*Nature Genetics*, in press 10.1101/2021.08.20.21262334). However, it should be noted, a systematic targeted gene-set analysis in that sample found significant overrepresentation of genes related to axon guidance but not for those involved in neuronal migration [48].

The Price et al. study also identified, at the less stringent p-value threshold (p~10^–5^), variants in or near genes implicated in neurodevelopmental disorders, particularly autism spectrum disorder (ASD) [53]. For example, *ANKS1B*, *CNTN4*, *RBFOX1*, *CSMD1*, and *ASTN2*. The study of the self-reported dyslexia in 23andme observed top associated SNPs in ASD-related genes. Although there is little evidence to support phenotypic comorbidity between ASD and RD, both are neurodevelopmental disorders and ASD involves deficits in language and communication skills [56, 57]. Further, there is some preliminary evidence of shared genetic risk factors: rare and *de novo* variants identified in ASD cases for genes that have been independently associated with RD (*PRTG*, *ROBO1,* and *KIAA0319L*) [58, 59], and altered neuronal migration has been suggested as a etiological mechanism in each condition [60–64]. While putative links between RD and DNM have received much attention in prior literature, few investigations to date have explored potential overlaps in neurobiological foundations of RD and ASD.

Given GWAS-based observations of associated SNPs in genes previously implicated in ASD and genes involved in NM/AG, along with investigations of DNM in previous candidate gene and postmortem studies, we wished to leverage this information to improve power to identify significant variants. Most existing GWAS samples are modest in size, with the exception of [47, 48], and relatively few findings reach statistical significance at the genome-wide level. To leverage power within available samples, we used Hypothesis-Driven (HD)–GWAS, which up-weights or prioritizes variants based on previously established genetic or biological hypotheses [65]. Statistical corrections are performed independently on the up-weighted and nonup-weighted groups, reducing the threshold for significance and increasing power. The HD-GWAS was primarily conducted on the Toronto sample [53]. We also wanted to examine the issues in the context of a larger dataset, so secondarily we used the meta-analysis of the GenLang Consortium [47]. A tertiary analysis was conducted using only those GenLang samples that were selected based on reading and/or language difficulties, to make it more comparable to the Toronto samples.

For our HD-GWAS of word reading, we formulated two separate hypotheses based on the results from previous GWAS (“conventional”, hypothesis-free GWAS) [53] and the literature. Specifically, we hypothesized that variants in (1) genes implicated in NM/AG and (2) genes implicated in ASD, would contribute to word reading.

## Methods

### Study populations and measures

To complete the HD-GWAS analyses, summary statistics from the Toronto conventional GWAS [53] and the meta-analysis of the GenLang Consortium were used [47]. The gene-set analyses made use of the raw genotypes of the Toronto sample and the summary statistics of GenLang. For both samples, quantitative measures of word reading were used as the phenotype.

#### Toronto sample

The Toronto sample has previously been described and is part of an ongoing genetic study of RD-selected families recruited at the Hospital for Sick Children [53, 56, 66]. At the time of analysis, the sample consisted of children identified with reading difficulties (*n* = 453) and both their unaffected and affected siblings (*n* = 171). Children were of European ancestry, as determined by PCA analysis. This choice aimed to reduce variation and create a more homogeneous population; however, it does not fully encompass the complex nature of allelic structure [67]. Children were excluded if there was evidence of other neurodevelopmental disorders, including ASD, apraxia, dyspraxia, central auditory processing disorder, stuttering, and psychiatric disorders, as well as medical conditions that would interfere with reading. Information on psychiatric and neurodevelopmental disorders was obtained using a structured parent interview [68] and a semi-structured teacher interview [69]. Children with ADHD, mild anxiety disorders, and speech/language difficulties were included.

The Toronto sample was measured for word reading using the Wide Range Achievement Test (WRAT) 3 [70]. The mean reading score was 88.4 [53]. Verbal assent and/or written consent was obtained from all children and parents. Procedural approval was given by the Hospital for Sick Children and University Health Network Research Ethics Boards.

#### The GenLang Consortium

The GenLang Consortium is a large international effort to study genetic contributions to traits related to speech, language, and reading (https://www.genlang.org). It does so through meta-analyses of these traits in population-based samples, as well as family-based and case-control cohorts [47]. For the purposes of the current study, the Toronto sample was analyzed separately, because the overlap between RD and neuronal migration/ASD was originally observed in this sample and formed the basis of the hypotheses being tested in this work.

The GenLang meta-analysis dataset used in this study consisted of 17 samples (*n* = 26,588) with individuals of European ancestry, as determined by PCA analysis (Table S1) [47]. Data quality control and filtering were performed by each individual sample before the meta-analysis [47]. We refer to this sample collectively as “The GenLang Cohort”.

In the GenLang Cohort, word reading was measured using a variety of standardized tests (depending on the individual sample) [47]. These phenotypic data were aligned across samples prior to the meta-analysis, as described in [47]. Consent was obtained from all participants in the GenLang Consortium and each individual sample’s institution-approved data use.

#### The GenLang selected subset

Five samples of the GenLang Cohort, selected for reading or language difficulties, were also examined (*n* = 4152, Table S1) as a subset. With exception of the SLIC cohort, all samples were selected for reading difficulties and participants were between the ages of 7 to 18. We refer to this collectively as the “GenLang selected subset”. In the family-based samples, phenotypic data was available both from probands and their siblings, regardless of affection status; therefore, these samples included a range of quantitative variation.

#### Data processing and GWAS of Toronto

Genotyping and quality control of the Toronto sample were previously described [53]. Briefly, DNA from each participant was genotyped on the OmniExpress platform and unobserved variants were imputed using the Michigan imputation server [71]. Quality control was conducted over numerous steps. Sex was checked using the heterozygosity of markers on the X chromosome. Sibling relationships were confirmed genetically and individuals with cryptic relationship were removed. In addition, variants with low imputation quality (R^2^ < 0.3), variants out of Hardy-Weinberg equilibrium (*p* < 0.0001), and variants with minor allele frequencies less than 5% were removed as well as samples with >2% missing genotypes and call rates <98%. After this filtering, 5.3 million SNPs were included in the analysis.

Because the Toronto sample included sibling pairs, the GWAS analysis was conducted using a linear mixed model in the R package ‘nlme’ to include a random effects term for family relationship [53]. Covariates for population structure (principal components) were also included as fixed effects. Only self-reported European Caucasian individuals were included in the analysis; verified by genotype. Principal components were generated in the program KING [72] and a Tracy-Widom statistic (EIGENSOFT Program) was used to determine significance [53].

#### Meta-analysis of the GenLang Cohort

Genotyping and quality control of the GenLang Cohort samples were previously described [47]. Individual cohort association analyses were performed with different tools, including SNPTEST [73], GEMMA [74], and PLINK [75] and included covariates for population structure (principal components) and family relationship.

A meta-analysis was performed on the samples using the program METAL [76]_,_ again using only individuals of European ancestry as determined by principal components. Effect size estimates were weighted with the inverse of the standard errors and genomic control on [47]. The GenLang selected subset underwent the same meta-analysis process. Summary statistics for the GenLang Cohorts were provided for use in this study after review and approval of the project by the coordinating board of the GenLang Consortium.

The Manhattan and quantile-quantile plots were generated using FUMA [77]. The regional association plot was examined in LocusFocus (https://locusfocus.research.sickkids.ca/) [78].

For both the GenLang Cohort and GenLang selected sample, only SNPs with a MAF ≥ 5%, and variants present in ≥50% of the total sample were used.

#### HD-GWAS

HD-GWAS serves as a powerful approach to GWAS by incorporating genetic or biological hypotheses based on the previously conducted research. This technique was developed and then tested by [65, 79–81]. Variants are divided into two strata: a stratum where it is hypothesized that variants are associated with the trait and a stratum where they are not. An estimated false discovery rate (FDR) is then calculated separately on each stratum. This prioritization leads to a less stringent type 1 error cut-off and increases the power to detect associated SNPs.

Although the Toronto sample did not originally meet SNP-based significance in the conventional GWAS, it did produce SNP level p-values of 10^–7^ and significant evidence by gene-based analysis. Thus, FDR correction was appropriate to increase power. The GenLang sample was a powerful sample with significant findings by conventional GWAS.

HD-GWAS was run using the stratified False Discovery Rate (sFDR) framework and program (http://www.utstat.toronto.edu/sun/Software/SFDR/index.html) [65, 79, 80]. As input, the program requires a SNP identifier, the p-value, and the weighting status (1- not up-weighted (not prioritized), 2- up-weighted (prioritized)). R (https://www.r-project.org/) and the command “merge ()” were then used to incorporate up-weighting information with summary statistics. Variants that were not in the up-weighted group formed the control stratum. The sFDR commands “-assoc” and “-SFDR” were used. The output included the FDR q-value and a sFDR q-value.

To test the first hypothesis, we up-weighted variants in genes implicated in NM/AG as well as growth cone formation. The gene list was made with AmiGO (http://amigo.geneontology.org), which uses the Gene Ontology (GO) database to annotate genes. The following search terms were used GO:0001764 neuron migration, GO:0007411 axon guidance, and GO:0030426 growth cone.

We broadened neuronal migration to include axon guidance and growth cone formation because many axon guidance molecules are pleiotropic, with diverse functions in multiple tissues and in the brain, including neuronal migration in the developing brain [82, 83] and growth cones are at the tips of the leading processes of migrating neurons and elongating axons [82, 83]. Genes in all three pathways have been implicated in disorders of neuronal migration, including periventricular nodular heterotopia [84], a neuronal migration disorder in which cortical development is compromised, leading to epilepsy and RD [85]. A total of ~115,000 variants in 351 genes were tested (Table S2). Within this gene list, we included the original RD-linked candidate genes that have been implicated in neuronal migration (*KIAA0319, DCDC2, DYX1C1,* and *ROBO1*).

To test the second hypothesis, we up-weighted variants in genes implicated in ASD using the gene list from the Simons Foundation Autism Research Initiative (SFARI) database (https://gene.sfari.org/database/human-gene/). More than half of these genes have been implicated in ASD through rare *de novo* mutations, or copy number variants (CNVs, syndromic or functional), but we also included those identified via genome-wide association studies. All categories were included, which at the time of the analysis consisted of 990 genes (Table S3) (~370,000 variants).

#### Gene-set analysis

The joint contribution of the genes annotated to each hypothesis was tested using gene-set analysis in MAGMA [86]. For the NM/AG and ASD hypotheses, the AMIGO and the SFARI gene lists, respectively, were used.

Gene-set analysis involves three steps, which were completed in MAGMA (https://ctg.cncr.nl/software/magma). For the first step genes were annotated to SNPs. Input for this step was raw genotype data for the Toronto sample and the reference data of the 1000 genomes project for the GenLang [87]. Second, individual gene analysis was completed to determine the association between each gene and the phenotype. For both samples, this step was performed using linear regression to compute a p-value for each gene. The input data were as follows. For the Toronto sample, the predictor variables were gene annotations from the previous step and principal components for population structure. The outcome variable was the word reading phenotype. For the GenLang datasets, the predictor variables were the gene annotations from the previous step and the outcome was the summary statistic p-values. Lastly, gene annotations were aggregated to their set and tested as a unit to see if they affected the trait. MAGMA took into consideration gene size, gene density, and allele count. The null hypothesis was that the genes tested as a group showed no greater association than a random set of genes.

#### Threshold for significance

The threshold for significance for the HD-GWAS and gene-set analyses was set at 2.5 × 10^–2^ (critical threshold 0.05/2 hypotheses). The Toronto sample formed the basis of our hypotheses and was corrected separately from the GenLang meta-analyses. The Toronto sample had no participant overlap with the GenLang Cohort.

## Results

### Conventional GWAS

The conventional GWAS for the Toronto and the GenLang Cohort have previously been published [47, 53]. The HD-GWAS analyses utilized the GenLang Cohort without the Toronto sample because the potential overlap between reading and neuronal migration/ASD was originally observed in that sample [53].

The GWAS of the GenLang selected subset, including only those GenLang samples that were recruited via probands with RD or language disorder, is a novel analysis that has not previously been described. The characteristics of cohorts in the selected subset are available within Supplemental Table 1 of Eising et al (2022). The Manhattan plot and quantile-quantile (Q-Q) plot for the selected subset are shown in Figs. S1–2. The *p* values depart from the expected line in the Q-Q plot at a *p* value of 10^−8^. The λ statistic was a value of 1.02.

For the conventional GWAS of the GenLang selected subset (no hypothesis tested), the top associated locus was on chromosome 21q21.1 in the intergenic region between genes *BTG3* and *C21orf91*. The most significant SNP was rs4818369 (Table 1, *p* = 2.37 × 10^–10^, threshold *p* ≤ 5 × 10^–8^; results at *p* < 10^–6^ shown in Table S4). The regional association plot is depicted in Fig. S3. Rs4818369 was not found to correlate with altered splicing or eQTLs. SNPs (*p*~10^–7^) in linkage disequilibrium (LD) (*r*^2^ > 0.3) with rs4818369 are eQTLs for the genes *BTG3* and *C21orf91* (GTEx v8, Table S5). For the conventional GWAS of the GenLang Cohort, this SNP did not show genome-wide significant association (*p* = 3.00 × 10^–3^).

**Table 1:** Conventional GWAS GenLang selected subset only.

SNP	Position	A1	Freq	Gene	Effect	SE	*P*	*N*
rs4818369	21:19055075	T	0.60	*BTG3-C21orf91*	−0.15	2.43 × 10^–2^	2.37 × 10^–10^	4152

*SNP* Single Nucleotide Polymorphism, Position chromosome: base pair (hg19), A1 Allele 1, Freq Allele Frequency, *SE* Standard Error, *P* *P* value, N Sample Size.

Only genome-wide significant results shown (significance threshold *p* = 5 × 10^−8^).

### HD-GWAS for the Toronto sample

For the HD-GWAS of the Toronto sample, no SNPs passed the threshold for significance when up-weighted based on the two hypotheses (threshold sFDR q ≤ 2.5 × 10^–2^; top 10 results in Tables S6–S7). However, when we tested the joint contribution of all genes in each of the individual hypotheses, we found the ASD-related gene-set was statistically significant (Table 2, *p* = 1.45 × 10^–2^, threshold *p* ≤ 2.5 × 10^–2^).

**Table 2:** Gene-set results for Toronto.

Hypothesis	Ngenes	Beta	SE	*P*
Neuronal Migration/Axon Guidance	327	0.066	0.048	0.08584
SFARI ASD	890	0.066	0.030	0.01448

*Ngenes* number of genes, *SE* Standard Error, *P* *P* value.

Significance threshold *p* ≤ 2.5 × 10^−2^.

### HD-GWAS for the GenLang Cohort

For the HD-GWAS of the GenLang Cohort, two loci on chromosome 1p31.3 and 20q13.33 in *DOCK7* and *CDH4*, respectively, passed the threshold for significance for the NM/AG hypothesis. The most significant SNP was rs1168041 for chromosome 1 and rs6089259 for chromosome 20 (Table 3, rs1168041 *p* = 6.61 × 10^–7^, rs6089259 *p* = 7.03 × 10^–6^, both sFDR *q* = 1.02 × 10^–2^, threshold sFDR *q* ≤ 2.5 × 10^–2^; results with *q* < 0.05 in Tables S8, S10. These SNPs were the top ranked SNPs by sFDR and in the prioritized group. Rs1168041 is an eQTL and splice quantitative trait locus (sQTL) for *DOCK7*, as are SNPs in LD (*r*^2^ > .03) with this marker (GTEx v8, Table S9. This SNP is in LD (*r*^2^ = .33) with the top SNP (rs11208009) in the original GenLang meta-analysis (22 samples, including the Toronto sample) located ~45 kb from *DOCK7* [47]. Rs11208009 is also an eQTL and sQTL for *DOCK7* (Eising et al. (2022) Supplementary) [47]. Rs6089259, intronic to *CDH4*, is not correlated with altered splicing or eQTLs according to available data.

**Table 3:** HD-GWAS results for the GenLang Cohort q < 0.05.

Hypothesis	SNP	Position	Gene	P GWAS	qSFDR
Neuronal Migration/Axon Guidance	rs1168041	1:62960250	*DOCK7*	6.61 × 10^–7^	1.02 × 10^–2^
	rs6089259	20:60246390	*CDH4*	7.03 × 10^–6^	1.02 × 10^–2^
	rs17158413	15:83235408	*CPEB1*	4.42 × 10^–5^	2.92 × 10^–2^

Total SNPs: 5,552,103.

Upweighted SNPs: Neuronal migration 115,448.

*SNP* Single Nucleotide Polymorphism, Position chromosome: base pair (hg19), P GWAS P-value in GWAS, qSFDR q-value from SFDR program.

Additional information including LD SNPs or SNPs in same gene region, rank and up-weighted status in the supplementary material.

Threshold for significance q ≤ 2.5 × 10^−2^.

For the gene-set analysis of the GenLang, no gene-sets passed the threshold for significance (threshold p ≤ 2.5 × 10^–2^, Table S11).

### HD-GWAS for the GenLang selected subset

For the HD-GWAS of the GenLang selected subset, the same locus and SNP (21q21.1, rs4818369) as the conventional GWAS passed the threshold for significance for both hypotheses (Table 4, *p* = 2.37 × 10^–10^, sFDR *q* < 9.00 × 10^–4^, 8.00 × 10^−4^, threshold sFDR *q* ≤ 2.5 × 10^–2^; results with *q* < 0.05 Tables S12–S13). This SNP was the top-ranked SNP by sFDR and not in the prioritized group, reflecting the robustness of the HD-GWAS [79]. Within the HD-GWAS and conventional GWAS of the GenLang selected subset, 14 and 18 SNPs, respectively, were previously identified with p < 10^–6^ in a prior GWAS analysis of word reading by Gialluisi et al. [50] (Tables S14–S15). The GenLang selected subset includes four cohorts which were included in the earlier Gialluisi et al. study [50], although at the time of that study the aforementioned cohorts were smaller in sample size than presently. The GenLang selected subset included one extra cohort (SLIC). The GenLang selected subset comprised 4152 individuals while Gialluisi included 3468 individuals.

**Table 4:** HD-GWAS results for GenLang selected subset only q < 0.05.

Hypothesis	SNP	Position	Gene	P GWAS	qSFDR
Neuronal Migration/Axon Guidance	rs4818369	21:19055075	BTG3-C21orf91**	2.37 × 10^–10^	9.00 × 10^–4^
	rs6090818	20.:46883131	LINC00494**	1.82 × 10^–7^	3.67 × 10^–2^
	rs6865160	5:31766737	*PDZD2*	2.82 × 10^–7^	4.10 × 10^–2^
SFARI ASD	rs4818369	21:19055075	BTG3-C21orf91**	2.37 × 10^–10^	8.00 × 10^–4^
	rs6090818	20.:46883131	LINC00494**	1.82 × 10^–7^	3.50 × 10^–2^
	rs6865160	5:31766737	*PDZD2*	2.82 × 10^–7^	3.92 × 10^–2^

Total SNPs: ~5,610,600.

Upweighted SNPs: Neuronal migration, 116,829; SFARI ASD 371,158.

*SNP* Single Nucleotide Polymorphism, Position chromosome: base pair (hg19), P GWAS P-value in GWAS, qSFDR q-value from SFDR program.

Additional information including LD SNPs or SNPs in same gene region, rank and up-weighted status in the supplementary material.

Significance threshold q ≤ 2.5 × 10^−2^.

**Intergenic and associated with word reading in Gialluisi et al., (6.79 × 10^−6^/3.14 × 10^−7^).

For the gene-set analysis of the GenLang selected subset, no gene-sets passed the threshold for significance (threshold *p* ≤ 2.5 × 10^–2^, Table S16).

## Discussion

Until recently, GWAS investigations of reading skills have identified few associated loci that passed the threshold for genome-wide significance. However, with the organization of large-scale collaborations, such loci are beginning to be found [47–50]. The number and size of cohorts characterized for reading-related traits has been a limiting factor. Therefore, methods that improve power to capitalize on existing samples are necessary to move the field forward in the short term. Overlap with top loci and genes known to be related to NM/AG and ASD susceptibility were observed in previous GWAS findings [47, 48, 53]. To test these observations, we used HD-GWAS, prioritizing variants in genes implicated in NM/AG or ASD susceptibility. We also tested the joint contribution of the genes and therefore the prioritization hypotheses themselves.

For the hypothesis testing the relationship to ASD, we did not identify significant SNPs by HD-GWAS. However, gene-set analysis determined that the hypothesis itself tested as the joint contribution of ASD-related genes was significant in the Toronto sample. There was no relationship in the GenLang meta-analysis. Previous GWAS studies that examined the relationship between ASD and RD [47, 48, 53] did not find genetic correlations using polygenic risk scores or LD Score Regression (LDSC). This may be because the GWAS for ASD to date are relatively small or because the cohorts were composed of diverse neurodevelopmental phenotypes as previously suggested [48]. Further, the PRS/LDSC analyses depend on summary statistics from GWAS analysis, which use only common variants. The SFARI dataset contains genes implicated in ASD by rare variants or CNV analyses, as well as genes identified from association studies of common variation. Thus, ASD-reading trait overlap may not be detectable via PRS methods because rare variants were not imputed in the GWAS analyses. Another possibility is that there may indeed be shared genes involved, but that the specific risk alleles are different for ASD and reading and are not in LD. HD-GWAS prioritizes genes irrespective of the contributing genetic variants and allows us to more formally quantify word reading–ASD associations in the Toronto sample, which was previously an observation [53].

For the Toronto sample, we excluded children with known or suspected ASD, or with a first-degree relative with ASD, or other global/intellectual disabilities. Overlap between reading- and ASD-associated genes likely stemmed from shared genetic risk for neurodevelopmental disorders, particularly those contributing to language-related difficulties, as opposed to global delays [56, 57].

For the NM/AG hypothesis, HD-GWAS using the GenLang Consortium data identified two associated loci with the top SNPs located in *DOCK7* and *CDH4*. *DOCK7*, Dedicator of Cytokinesis 7, is involved in axon formation and neuronal polarization [55]. The top marker, rs1168041, is an eQTL and sQTL for *DOCK7*. SNPs distal to *DOCK7* were previously identified as significantly associated with word reading in the GenLang Consortium meta-analysis with the top SNPs also eQTLs and sQTLs for *DOCK7* [47]. *CDH4*, Cadherin 4, encodes a cell-cell adhesion glycoprotein thought to play a role in cortical development and neuronal outgrowth [88]. *CDH4* has not been implicated in RD traits in previous studies.

Our HD-GWAS analyses using the selected GenLang Consortium data identified a significant locus associated with word reading for both hypotheses, indicating that the result is robust and found even though this locus is not within the upweighted SNP sets. The top associated markers (rs4818369) are located between the genes *BTG3* and *C21orf91*, and LD SNPs are eQTLs for both genes which are credible candidates for involvement in RD. *BTG3*, BTG Anti-Proliferation Factor 3, is implicated in neurogenesis [89, 90], and *C21orf91*, also known as *EURL* (Early Undifferentiated Retina and Lens), is implicated in oligodendroglia differentiation, influencing the cell’s capacity to mature and to myelinate axons [91].

The results of our HD-GWAS and gene-set analyses, although statistically significant in the separate samples, were not replicated across the Toronto and GenLang datasets. Thus far, few associated loci have been found to overlap between samples of self-reported dyslexia, quantitative measures of reading in population-based, and RD-selected cohorts [50, 53]. Nonetheless, these same studies yield evidence of considerable genetic overlap between quantitative measures of reading and self-report dyslexia, as shown by genetic correlation analyses using the GWAS data (*r*_*g*_ = −0.71). The lack of overlap for individual loci may simply be a function of power, which could change with larger sample sizes, but a role for differences in ascertainment cannot be ruled out. Clinically ascertained GWAS samples may be enriched for participants with comorbid disorders as individuals with multiple conditions are more likely to come to clinical attention (Berkson’s Bias) [92], increasing the identification of genes related to those disorders. Alternatively, clinical studies may screen for, and exclude participants with, comorbid or medical conditions or environmental factors that would interfere with reading acquisition. These exclusions could alter the composition of the sample compared to population-based samples and possibly influence gene findings.

In summary, through an HD-GWAS framework, we identified significant associations with reading skills. We also found that genes related to ASD risk contribute to RD in the Toronto sample. Our findings support two core features of the HD-GWAS framework. First, this framework is robust to stratifying misspecification of up-weighted variants (i.e., less than ideal hypotheses [65]). We demonstrated this feature when using HD-GWAS we identified the same chromosomal 21 SNPs from conventional GWAS, even though they were not in the up-weighted group for the GenLang selected subset. Second, this framework increases power to identify genes within hypothesized pathways/mechanisms compared to unstratified approaches. We illustrated this feature when we found that the ASD-related gene-set contributed to reading and identified loci upweighted in the NM/AG hypothesis. Future studies involving larger GWAS samples ascertained through reading and language disorders may help to elucidate shared genetic mechanisms between RD and ASD.

## Supplementary information

S. Fig 1: Manhattan Plot

S. Fig 2: Quantile-Quantile (Q-Q) Plot

S. Fig 3: Regional Association Plot

Supplementary Tables

## Data Availability

Summary statistics for the Toronto sample and the GenLang sample used in this study are available upon application to the GenLang Consortium (http://www.genlang.org) and review of the proposal. To download summary statistics for the entire GenLang (not the sample specific to this study), use http://www.genlang.org or the public GWAS catalogue.
