# Supplementary material for: Hypothesis-driven genome-wide association studies provide novel insights into genetics of reading disabilities
Source: Transl Psychiatry. 2022 Nov 29;12:495. doi: 10.1038/s41398-022-02250-z (PMC9709072; doi:10.1038/s41398-022-02250-z)

**S. Figure 1: Manhattan plot.** Manhattan plot for the GenLang Consortium Selected Subset. Genome-wide threshold for significance p<5 x 10^-8^ (<https://fuma.ctglab.nl/>).

**S. Figure 1:**

Manhattan Plot for the Conventional GWAS of the GenLang Consortium Selected Subset


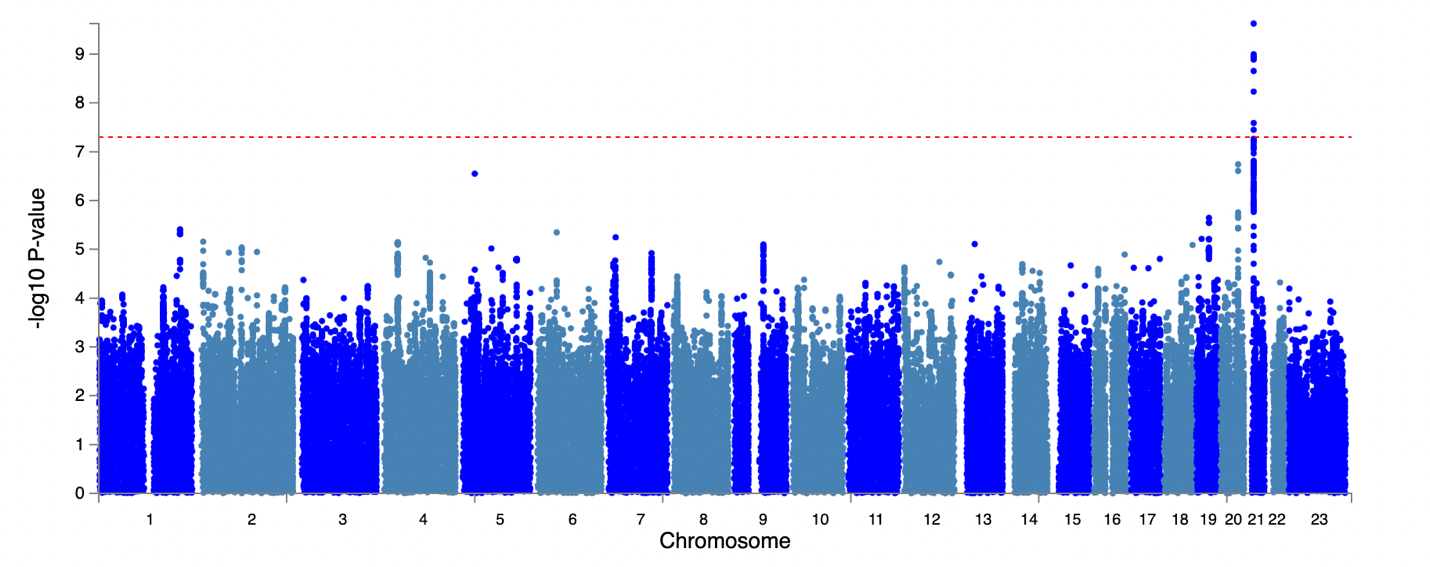

Supplement: Supplementary file 1 — S. Fig 1: Manhattan Plot [file 41398_2022_2250_MOESM1_ESM.docx]
