# Supplementary material for: Hypothesis-driven genome-wide association studies provide novel insights into genetics of reading disabilities
Source: Transl Psychiatry. 2022 Nov 29;12:495. doi: 10.1038/s41398-022-02250-z (PMC9709072; doi:10.1038/s41398-022-02250-z)

**S. Figure 2: Quantile-Quantile (Q-Q) Plot.** Q-Q plot for the GenLang Consortium Selected Subset. Genome-wide threshold for significance p<5 x 10^-8^ (https://fuma.ctglab.nl/).

**S. Figure 2:**

Q-Q Plot for the GenLang Consortium Selected Subset


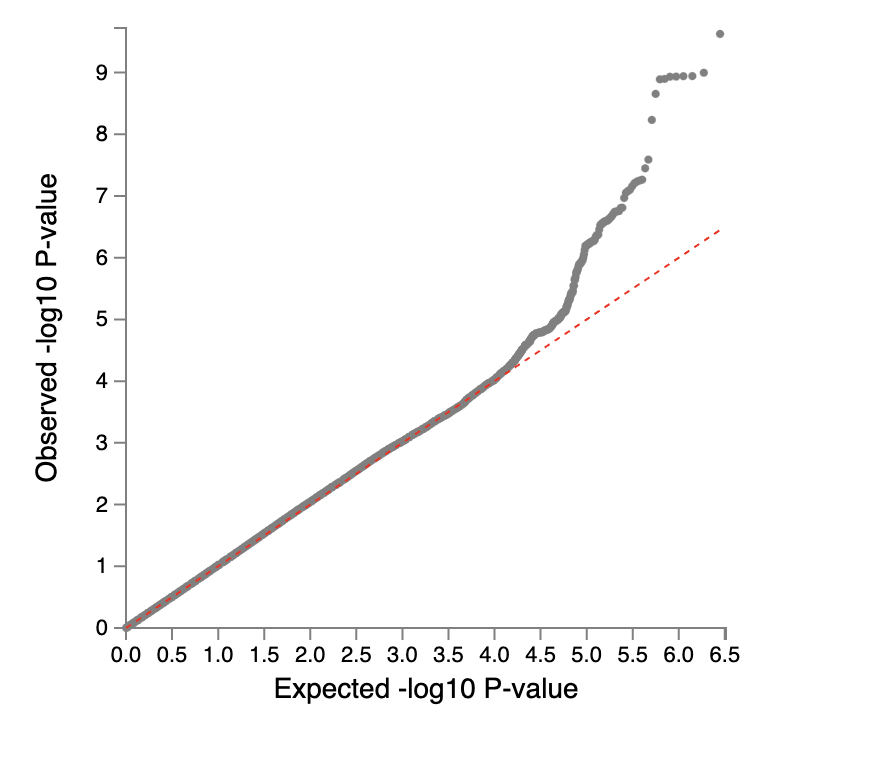

Supplement: Supplementary file 2 — S. Fig 2: Quantile-Quantile (Q-Q) Plot [file 41398_2022_2250_MOESM2_ESM.docx]
