# Supplementary material for: Hypothesis-driven genome-wide association studies provide novel insights into genetics of reading disabilities
Source: Transl Psychiatry. 2022 Nov 29;12:495. doi: 10.1038/s41398-022-02250-z (PMC9709072; doi:10.1038/s41398-022-02250-z)

**S. Figure 3: Regional Association Plot.** Regional association plot for the GenLang Selected Subset for chromosome 21 (rs4818369). The circle colours represent the LD correlations (*r*^2^). Grey is no LD information, dark blue is less than 0.2, light blue is 0.2-0.4, green is 0.4-0.6, orange is 0.6-0.8, red is higher than 0.8, and purple is the lead SNP.

**S. Figure 3:**
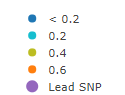


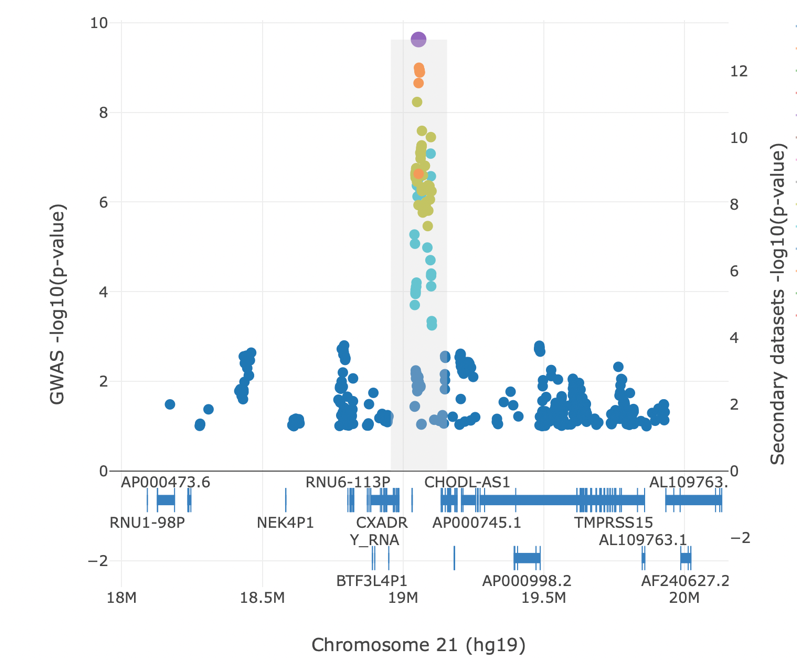

Supplement: Supplementary file 3 — S. Fig 3: Regional Association Plot [file 41398_2022_2250_MOESM3_ESM.docx]
